# Backstabbing

**DOI:** 10.4103/0974-2700.76813

**Published:** 2011

**Authors:** Kenji Kawamukai, Filippo Antonacci, Sergio Nicola Forti Parri, Salomone Di Saverio, Maurizio Boaron

**Affiliations:** Department of Thoracic Surgery, Maggiore and Bellaria Hospitals, Bologna, Italy; 1 Department of Emergency Surgery, Maggiore Hospital, Bologna, Italy

Sir,

Although knife stabbing is frequently seen in most trauma centers, retained knife blade is an uncommon occurrence. Withdrawal of the impacted knives represents a great challenge, since unplanned extraction can result in catastrophic outcomes: massive hemorrhage, hemodynamic deterioration, and death.

In the case we report, even though clinically impressive, no major complications occurred given that the blade did not impact any vital organ. Nevertheless, an exhaustive diagnostic evaluation and a carefully operative planning are essential for the successful management of this circumstance.

A 39-years-old caucasian male walked into emergency department after being stabbed in the back by a rival during a lovers quarrel in a club. On admission to the emergency room, the patient was in a stable hemodynamic condition. He was talking normally and was eupnoic. Chest X-ray did not show hemothorax or pneumothorax without mediastinal widening or emphysema. However, the shadow of the knife was mid-thoracic, projected toward the heart, major vessels and trachea.

**Figure 1:**
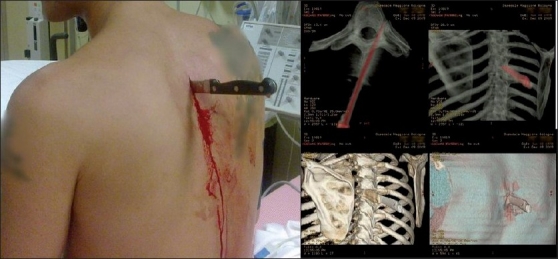
Clinical presentation and CT 3D reconstruction

Given his clinical stability, the patient was brought to computed tomography (CT) scan leaving the blade in place. 3D and multiplanar reformations (MPR) CT [Figure 1] excluded penetration of the pleura or mediastinum. The knife edge impacted the vertebras between T4 and T5, wedging into the left costovertebral joint. The patient was transferred to the operating theater and the knife was removed under sedation. The procedure did not present any complications He was admitted for observation and received antibiotics. He was discharged the following day and his further course has been uneventful.

Retained knife blade, although clinically spectacular, has been rarely reported in the medical literature and most trauma centers have limited experience with this kind of injury.[1]

Clinical examination should systematically include the assessment of the extent and depth of the entry wound to speculate any possible specific organ and neurovascular injuries. Patients with retained blades and major vessel injuries can be stable hemodynamically on admission only because the blade acts as a plug and prevents hemorrhage. Spiral CT is essential to define the relationship between the retained knife and major visceral and vascular structures. In case of inconclusive CT scans, if uncertainties of blood vessels injury persist, angiography is indicated.[1]

Every case of retained blade injury must be considered as a unique event. Its complexity gives reason for the absence of published guidelines regarding a standardized clinical and operative management of retained blades.

Hemodynamic collapse following unplanned extraction of retained knives by the inexperienced medical personnel is reported in the literature.[2] Although there is no currently consensus whether retained blades should be withdrawn in the emergency department or in the operating room, the procedure should always be performed with the availability of surgical staff, anesthesia team and adequate instrumentation to handle every possible surgical complication and resuscitation necessity.

All these measures are not a guarantee for success, but they can significantly limit catastrophic consequences following simple withdrawal.
